# Medicare Skilled Home Health Care Quality: Are We Measuring What Matters for People Living With Dementia?

**DOI:** 10.1093/geroni/igaf122.284

**Published:** 2025-12-31

**Authors:** Jennifer Reckrey, Bian Liu, Arushi Arora, Christine Ritchie, Bruce Leff, Abraham Brody, Julia Burgdorf, Katherine Ornstein

**Affiliations:** NYU Grossman School of Medicine, New York, New York, United States; Icahn School of Medicine at Mount Sinai, New York, New York, United States; Icahn School of Medicine at Mount Sinai, New York, New York, United States; Massachusetts General Hospital, Boston, Massachusetts, United States; Johns Hopkins University School of Medicine, Baltimore, Maryland, United States; NYU Rory Meyers College of Nursing, New York, New York, United States; VNS Health, New York, New York, United States; Johns Hopkins University, Baltimore, Maryland, United States

## Abstract

People living with dementia frequently use Medicare skilled home health care to meet their care needs at home, but little is known about variation in care quality for this population. Using 100% 2021 Medicare Claims data, we examined receipt of high-quality home health as determined by the 1) Quality of Patient Care Star Rating and 2) Patient Survey Star Rating among fee-for-service beneficiaries with dementia. Of the over 1.6 million Medicare fee-for-service beneficiaries with dementia in 2021, 34% used home health. We found significant county-level variability in utilization of high-quality home health. Within a given county, people living with dementia were consistently less likely to use high-quality home health agencies, regardless of whether quality was measured using the Quality of Patient Care Star Rating or the Patient Survey Star Rating. These findings highlight disparities in care quality received by persons living with dementia and underscore the limited utility of existing home health quality measures for this population. Specifically, quality measures focusing on functional improvement are unlikely to meaningfully reflect care quality for people living with dementia experiencing progressive functional decline as part of their disease. Additional work is needed to optimize care quality and customize quality measures to the unique care needs and experiences of people living with dementia.

